# Supplementary figures and images for: Green Synthesized Polymeric Iodophors with Thyme as Antimicrobial Agents
Source: Int J Mol Sci. 2024 Jan 17;25(2):1133. doi: 10.3390/ijms25021133 (PMC10815993; doi:10.3390/ijms25021133)

EDS Layered Image 6

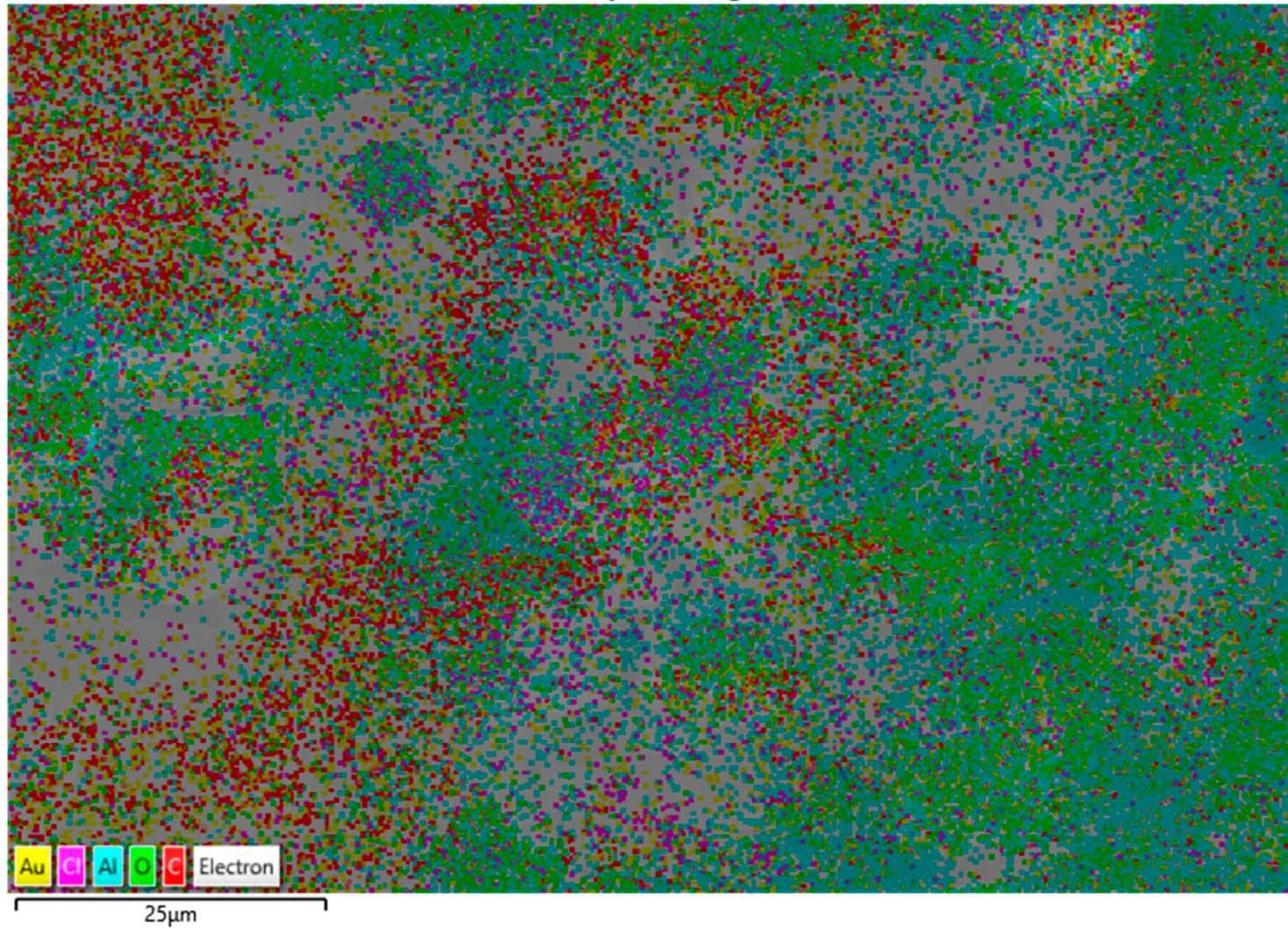

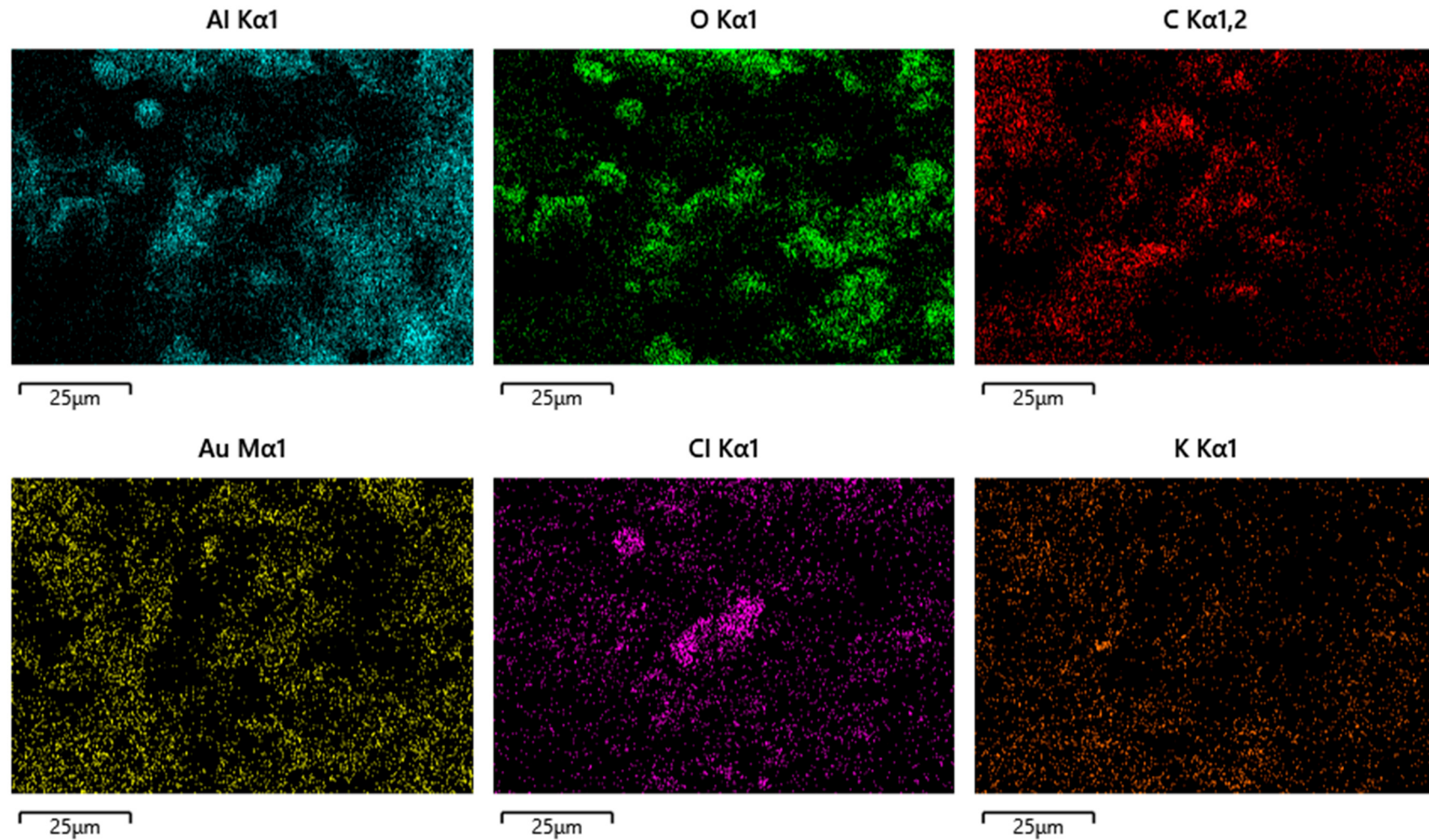

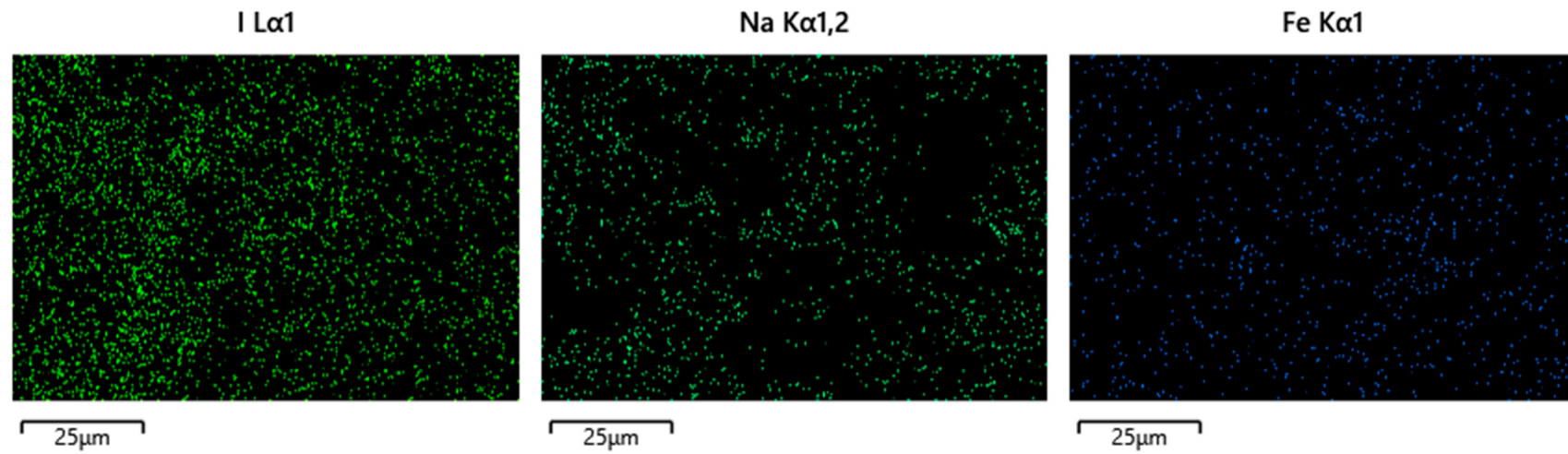

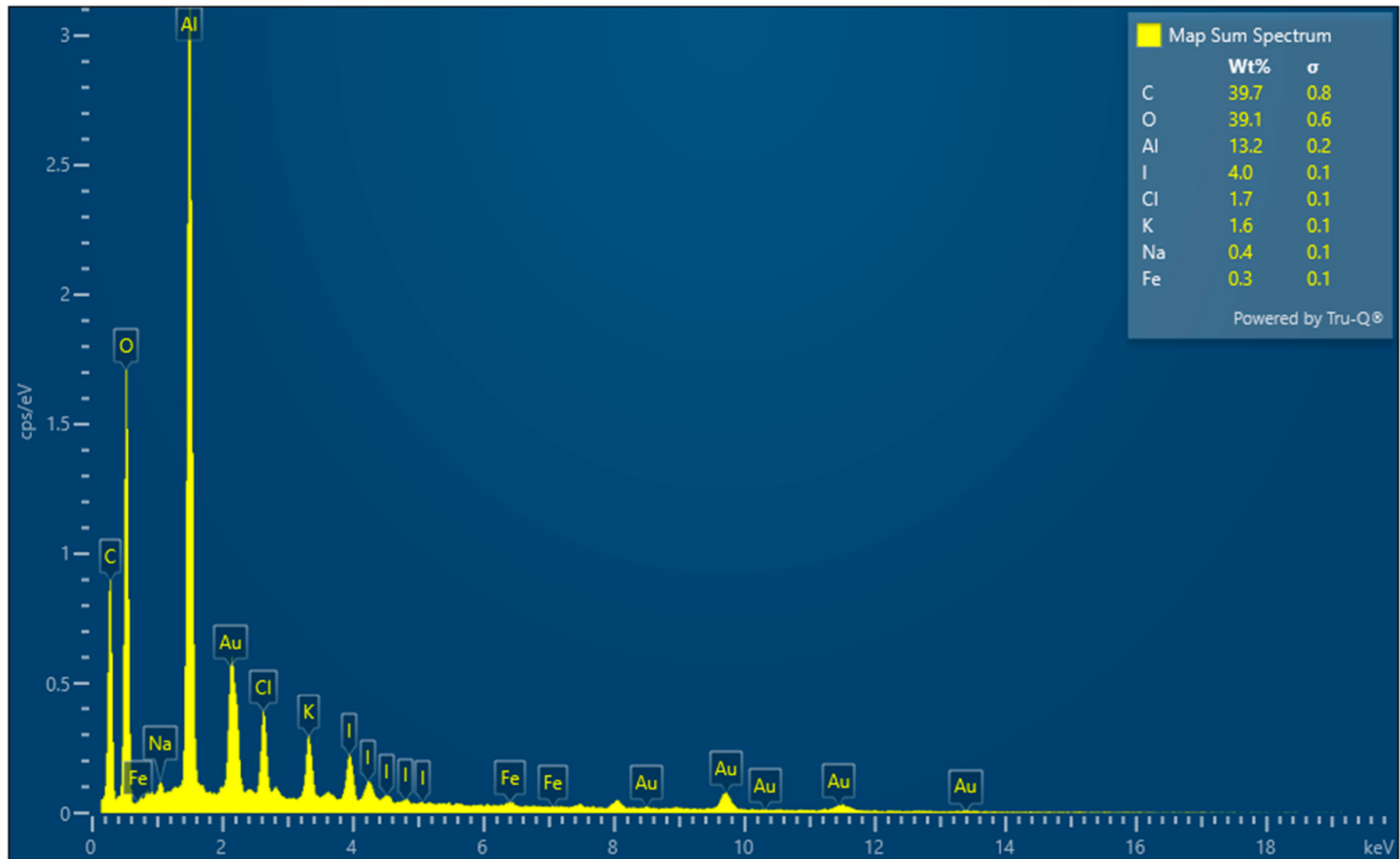

Supplement: Supplementary file 1 [file ijms-25-01133-s001.zip › Supplementary File S1-EDS-AV-PVP-Thyme-I2.pdf]

EDS Layered Image 11

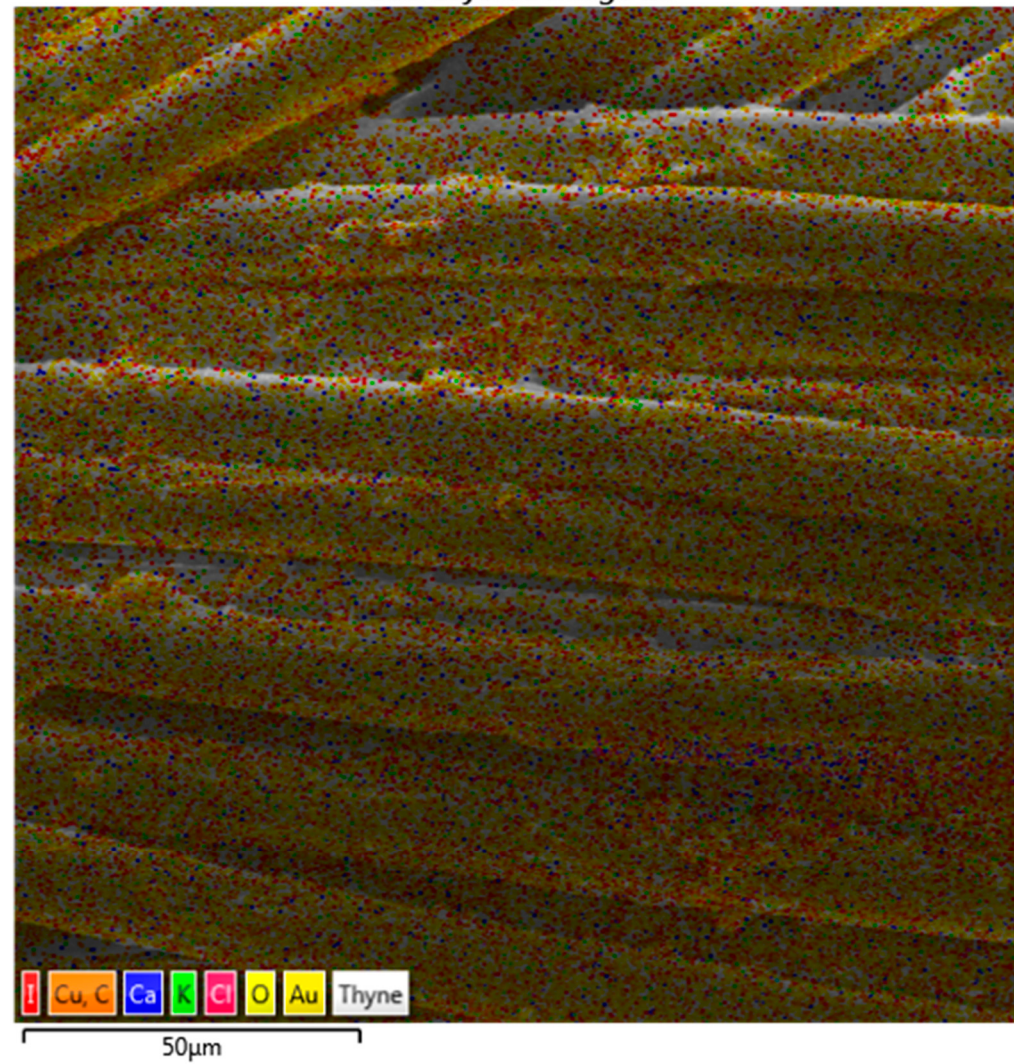

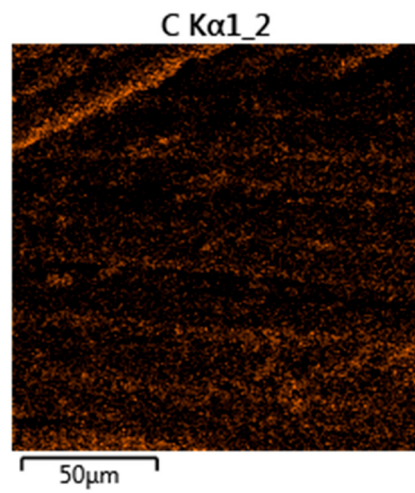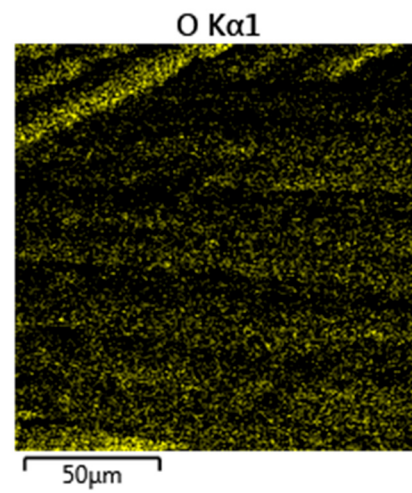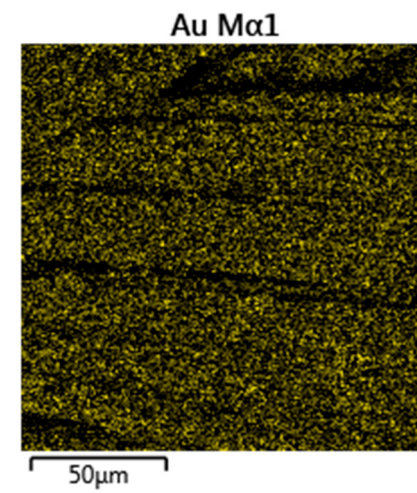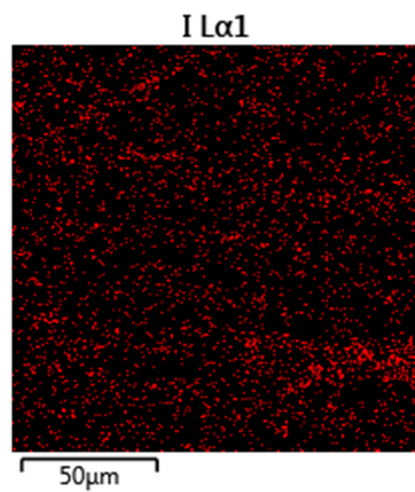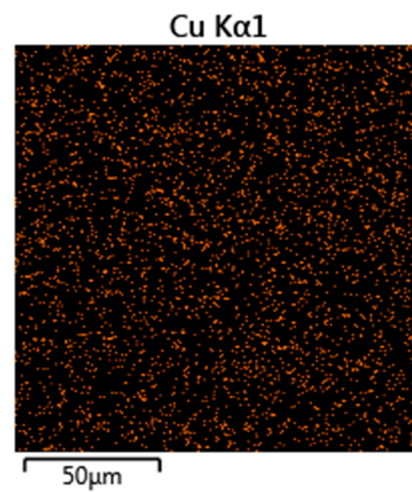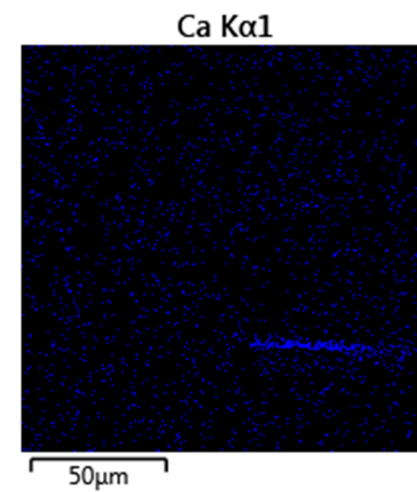

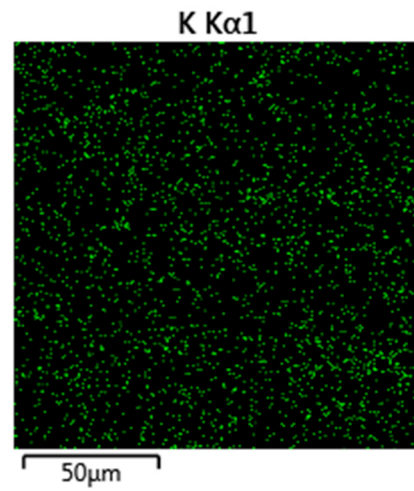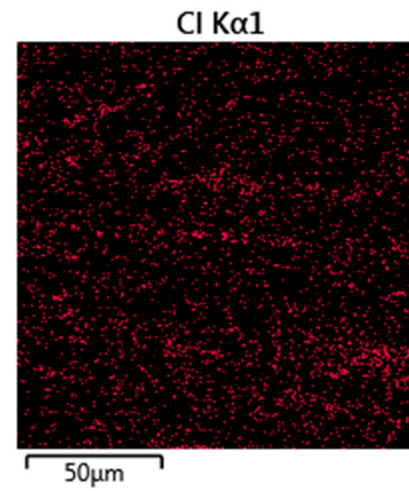

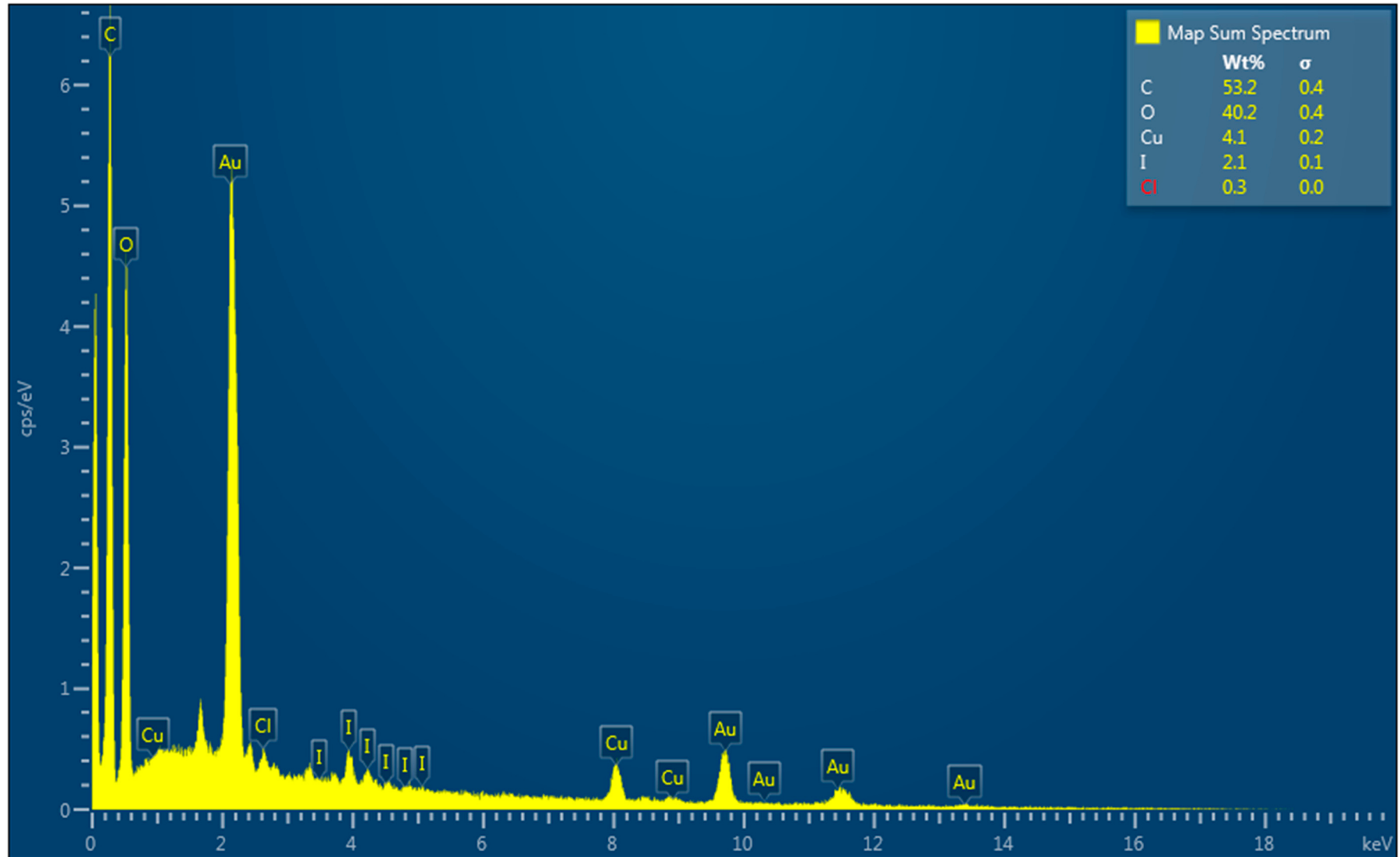

Supplement: Supplementary file 1 [file ijms-25-01133-s001.zip › Supplementary File S2-EDS-AV-PVP-Thyme-I2 SUTURE.pdf]

EDS Layered Image 2

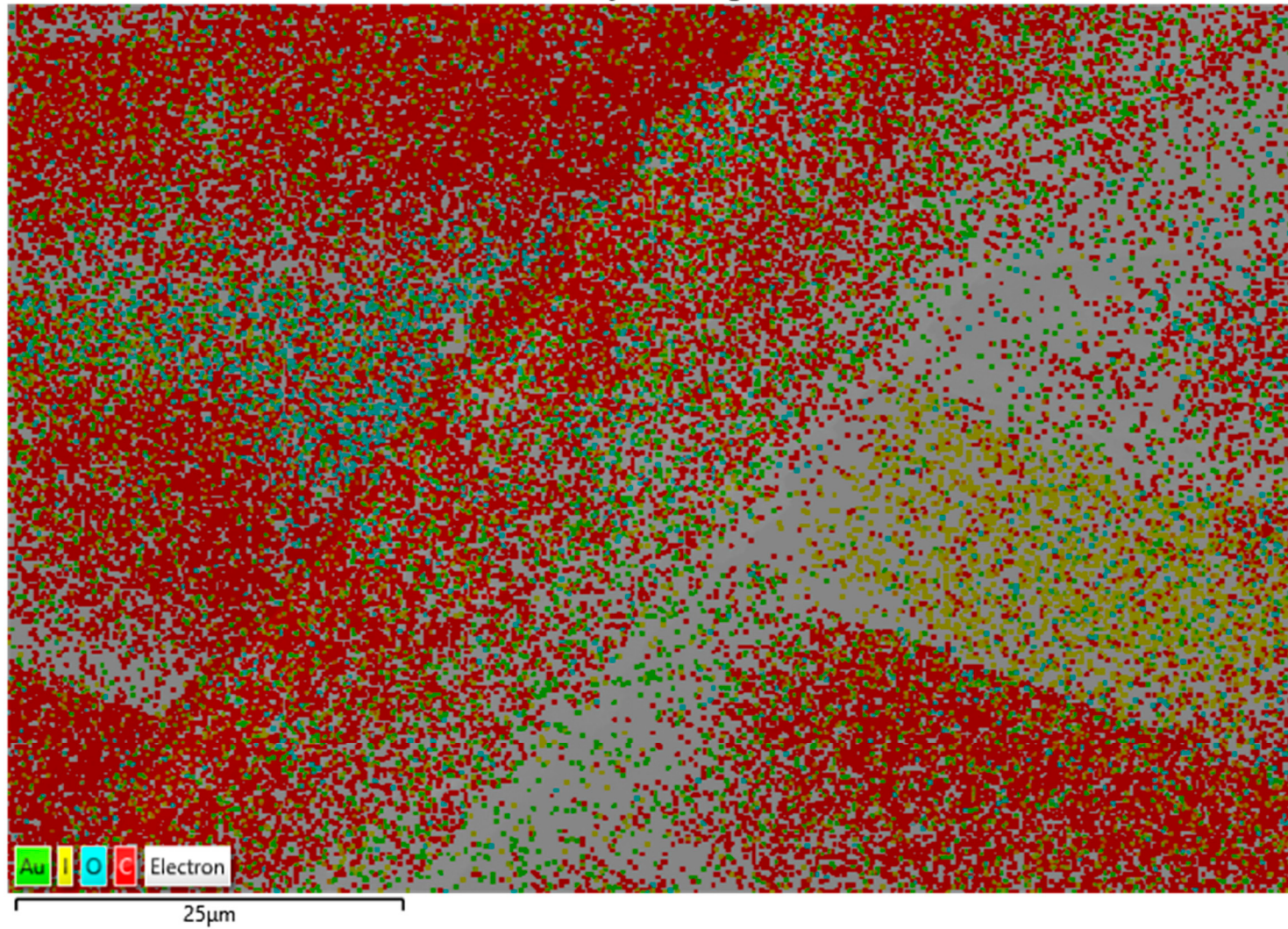

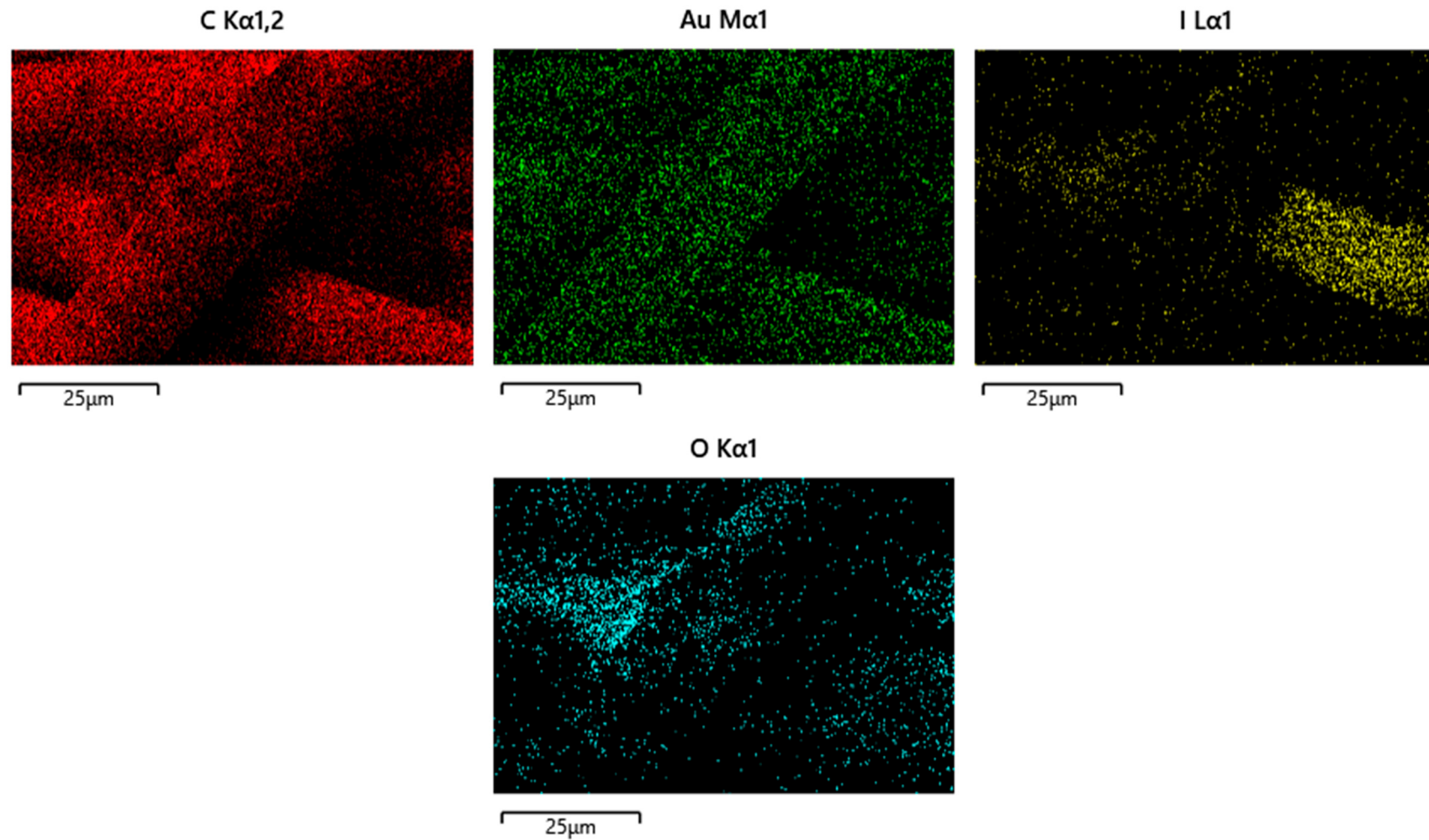

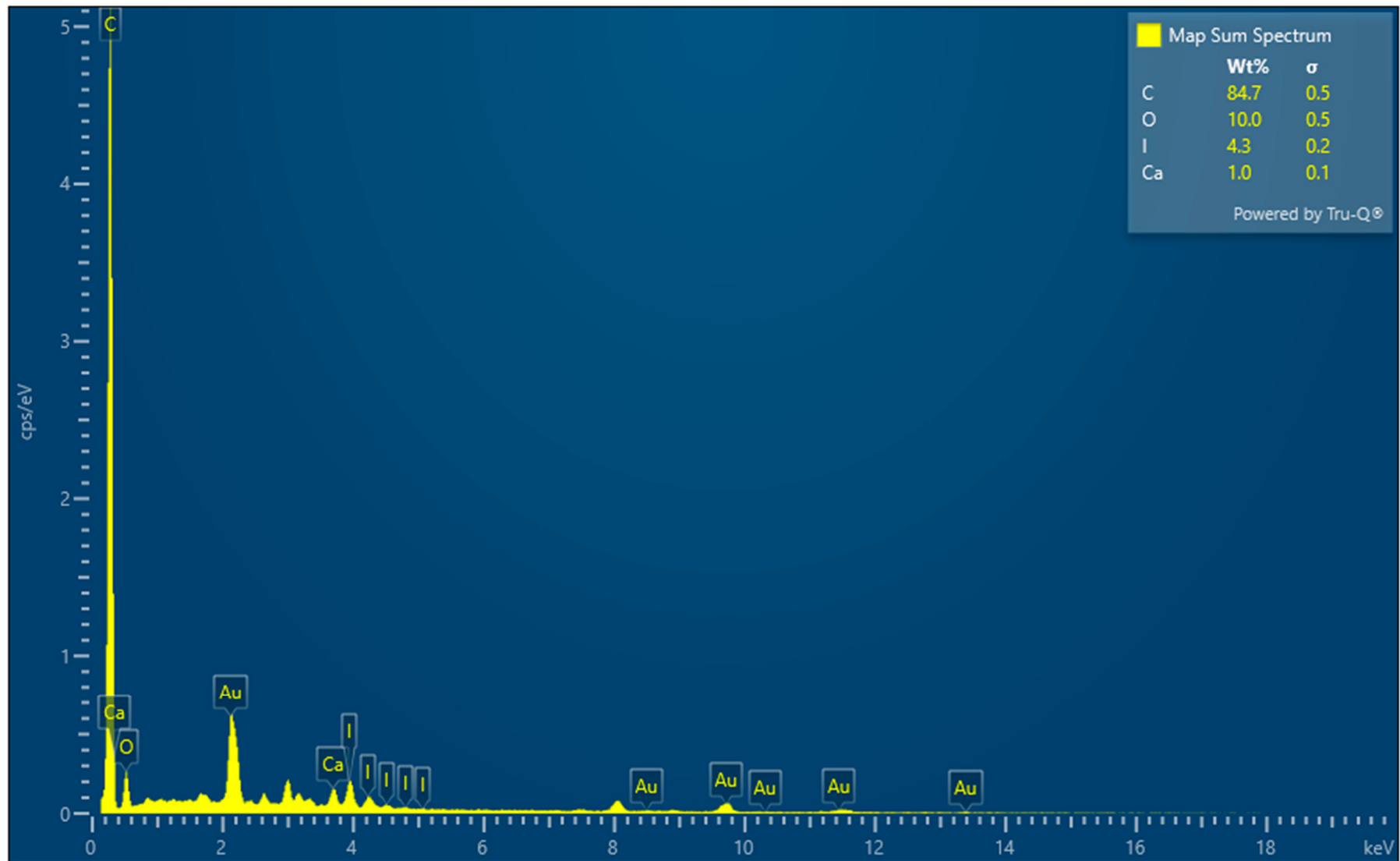

Supplement: Supplementary file 1 [file ijms-25-01133-s001.zip › Supplementary File S3-EDS-Thyme 4 mask dense-white inner layer-near-4.3.pdf]

EDS Layered Image 4

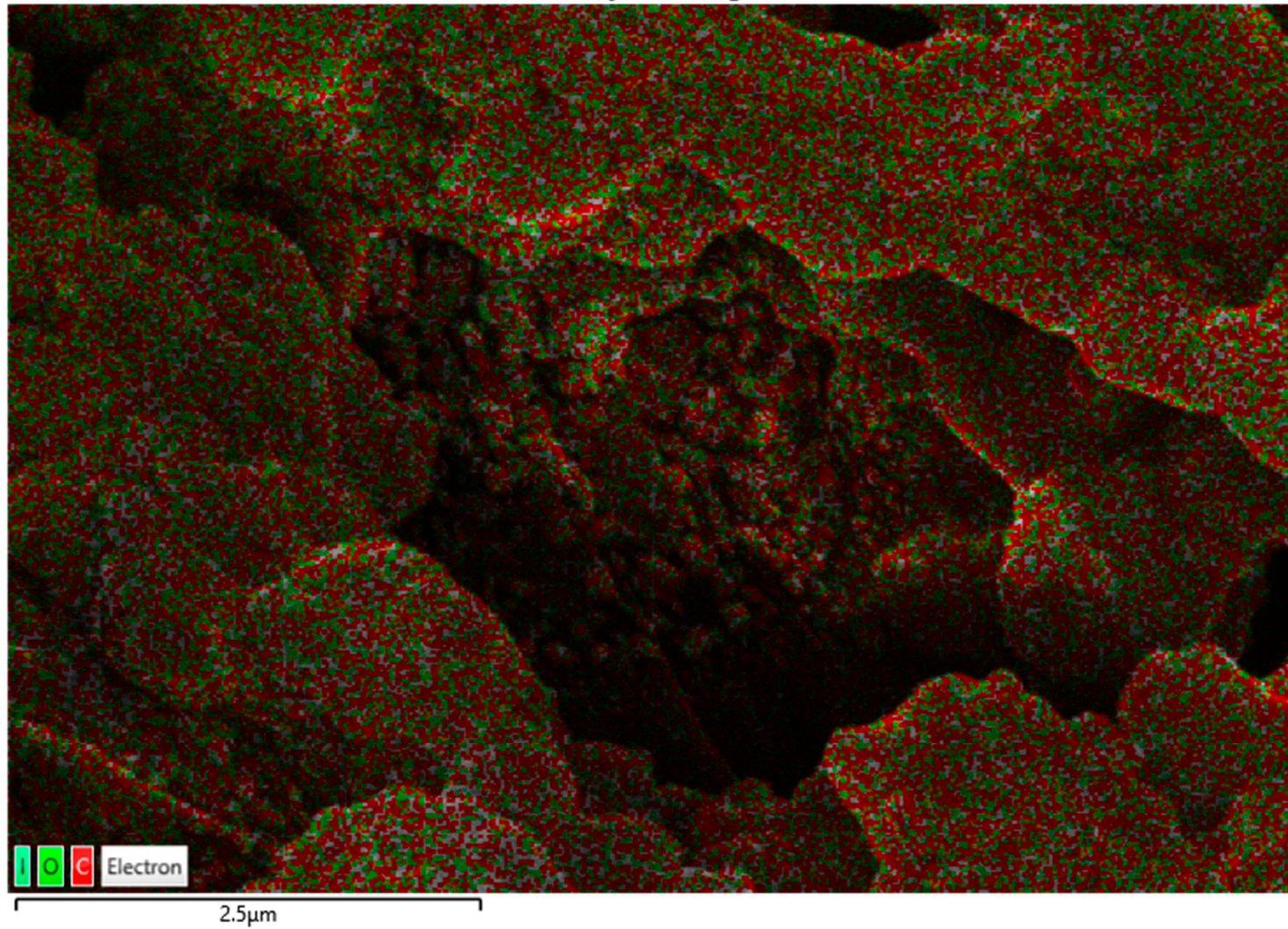

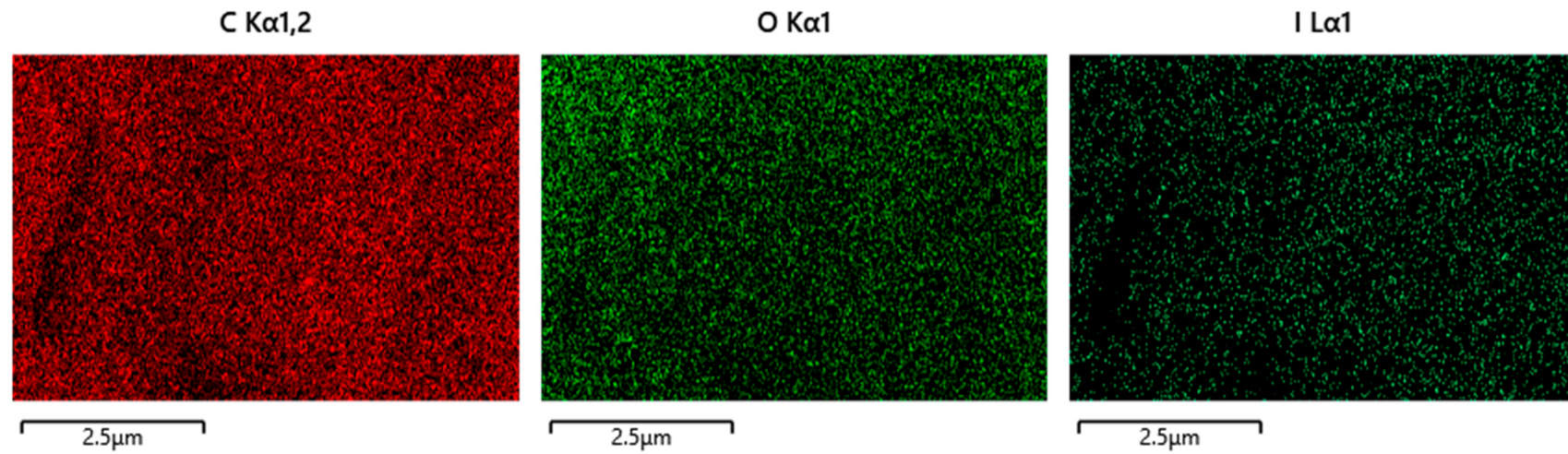

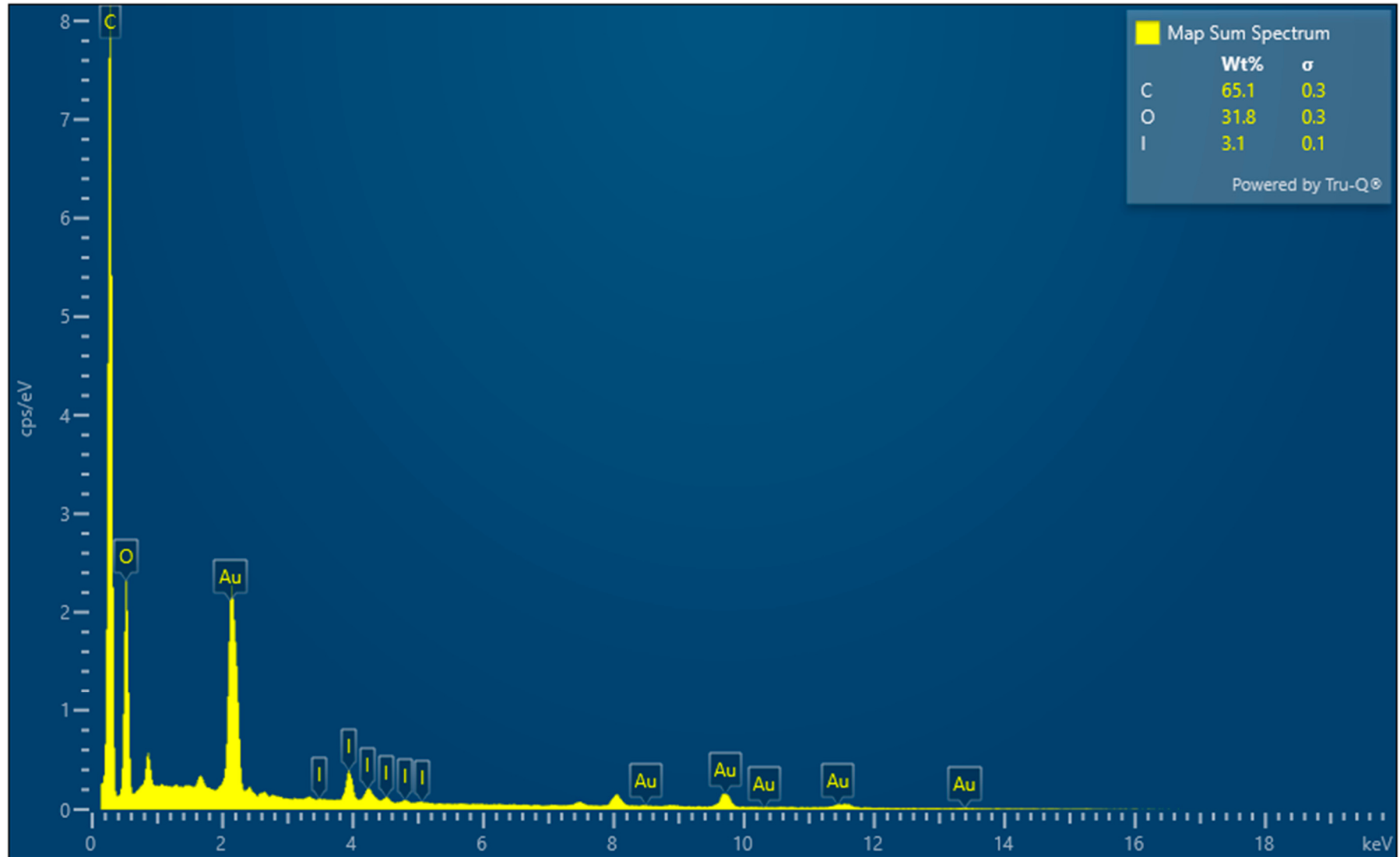

Supplement: Supplementary file 1 [file ijms-25-01133-s001.zip › Supplementary File S4-EDS-AV-PVP-Thyme-I2 Bandage near.pdf]
